# Homogeneous Metastable Hexagonal Phase Iridium Enhances Hydrogen Evolution Catalysis

**DOI:** 10.1002/advs.202206063

**Published:** 2023-02-12

**Authors:** Shize Geng, Yujin Ji, Jiaqi Su, Zhiwei Hu, Miaomiao Fang, Dan Wang, Shangheng Liu, Ling Li, Youyong Li, Jin‐Ming Chen, Jyh‐Fu Lee, Xiaoqing Huang, Qi Shao

**Affiliations:** ^1^ College of Chemistry Chemical Engineering and Materials Science Soochow University Jiangsu 215123 China; ^2^ College of Energy Xiamen University Xiamen 361102 P. R. China; ^3^ Institute of Functional Nano & Soft Materials (FUNSOM) Soochow University Jiangsu 215123 P. R. China; ^4^ Max Planck Institute for Chemical Physics of Solids Nothnitzer Strasse 40 01187 Dresden Germany; ^5^ College of Energy Soochow University Jiangsu 215123 P. R. China; ^6^ National Synchrotron Radiation Research Center 101 Hsin‐Ann Road Hsinchu 30076 Taiwan; ^7^ State Key Laboratory of Physical Chemistry of Solid Surfaces College of Chemistry and Chemical Engineering Xiamen University Xiamen 361005 China

**Keywords:** electrocatalysis, hydrogen evolution reaction, iridium, metastable phase

## Abstract

Catalytic reactions are surface‐sensitive processes. Fabrication of homogeneous metastable metals can be used to promote phase‐dependent catalytic performance; however, this has been a challenging task. Herein, homogeneous metastable hexagonal close‐packed (hcp) Ir is epitaxially grown onto metastable phase hcp Ni, as demonstrated using spherical aberration electron microscopy. The as‐fabricated metastable hcp Ir exhibits high intrinsic activity for the alkaline hydrogen evolution reaction (HER). In particular, metastable hcp Ir delivers a low overpotential of 17 mV at 10 mA cm^−2^ and presents a high specific activity of 8.55 mA cm^−2^ and a high turnover frequency of 38.26 s^−1^ at −0.07 V versus the reversible hydrogen electrode. Owing to its epitaxially grown structure, metastable hcp Ir is highly stable. Theoretical calculations reveal that metastable hcp Ir promotes H_2_O adsorption and fast H_2_O dissociation, which contributes to its remarkable HER activity. Findings can elucidate the crystal phase‐controlled synthesis of advanced noble metal nanomaterials for the fundamental catalytic applications.

## Graphical abstract

Metastable hcp Ir is synthesized for the first time by epitaxially growing Ir on hcp Ni. This is a remarkable breakthrough in the field of metastable noble metals. Hcp Ir‐Ni exhibits excellent hydrogen evolution reaction activity, outperforming most electrocatalysts reported to date.

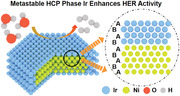

## 1 Introduction

Owing to the ongoing global energy and pollution crises, researchers have focused on developing highly efficient catalysts for renewable energy conversion reactions.^[^ ^1^, ^2^ ^]^ Metastable phase materials are commonly used as catalysts owing to the high‐energy structure of their unequilibrated metal surfaces.^[^ ^3^, ^4^, ^5^ ^]^ The quest for new polymorphisms of current metals is a primary goal in the field of material science, because catalytic performance is closely related to atom ordering.^[^ ^6^, ^7^, ^8^, ^9^ ^]^ The current stable polymorphisms of the bulk Pt group metals are face‐centred cubic (fcc) for Ir, Pt, Pd, and Rh and hexagonal close‐packed (hcp) for Os and Ru.^[^ ^10^, ^11^, ^12^ ^]^ Therefore researchers should explore new polymorphisms for the Pt group metals.

Among Pt group metals, Ir has attracted considerable attention because it is a promising catalyst for electrocatalysis, organic reactions, and photoredox applications.^[^ ^13^, ^14^, ^15^, ^16^ ^]^ However, owing to the high price and scarce resources of Ir, it is critical to design catalysts with ultralow Ir content.^[^ ^17^, ^18^ ^]^ Ir is the most stable noble metal, and its only thermodynamically stable phase, the fcc phase, exhibits a characteristic packing ordering of “ABCABC” along the close‐packed [111] direction.^[^ ^19^ ^]^ Owing to the intrinsic properties of fcc Ir, preparation of metastable Ir with good catalytic performance is challenging. For nanomaterials, surface energy is the predominant factor that determines total energy. This favors the synthesis of metals with new atomic packings, which are different from those of bulk metals.^[^ ^20^, ^21^ ^]^

Inspired by these possibilities, herein, we fabricated homogeneous metastable hcp Ir. Thorough experiments demonstrated that the packing sequence of Ir along the close‐packed [001] direction became “ABAB”. Therefore, Ir exhibited homogeneous metastable hexagonal ordering (Figure S1, Supporting Information). As a proof‐of‐concept application, the surface of the as‐synthesized metastable hcp Ir presented remarkable activity for the hydrogen evolution reaction (HER) in alkaline media. The overpotential of metastable hcp Ir, which was only 17 mV at the current density of 10 mA cm^−2^, is one of the lowest reported overpotentials. Moreover, metastable hcp Ir exhibited high intrinsic activity with a high turnover frequency (TOF) of 38.26 s^−1^ at −0.07 V vs the reversible hydrogen electrode (RHE), which was considerably higher than that of fcc Ir. The high stability of hcp Ir was attributed to the experimental conditions during fabrication using an epitaxial growth method. Theoretical calculations revealed that the co‐effect of the strong H_2_O adsorption and fast H_2_O dissociation over metastable hcp Ir improved the HER activity of hcp Ir in alkaline media.

## 2 Results and Discussion

**Figure 1:**
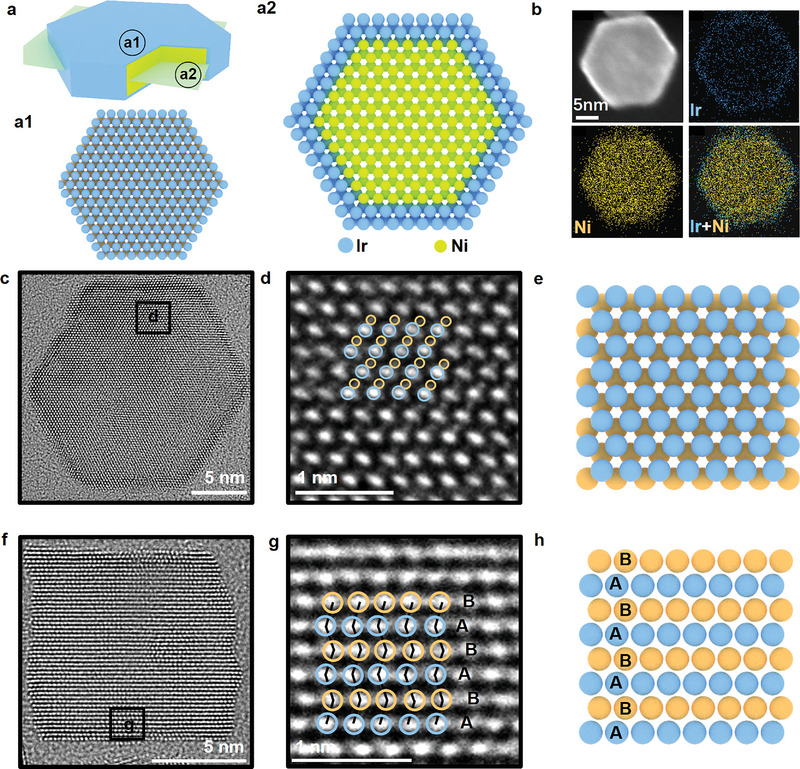
Electronic structure characterization of hcp Ir‐Ni. a) A model of hcp Ir‐Ni nanoplate from (a1) vertical view and (a2) perspective view. b) TEM‐EDX mapping of Ir and Ni, confirming a core‐shell structure. c) The spherical aberration corrected HAADF‐STEM image of hcp Ir‐Ni from [001] direction. d) High‐resolution HAADF image taken from the selected area in (c). e) A model of hcp Ir‐Ni from [001] direction. f) The spherical aberration corrected HAADF‐STEM image of hcp Ir‐Ni from [100] direction. (g) High‐resolution HAADF image taken from the selected area in (f). h) A model of hcp Ir‐Ni from [100] direction.

The structural model of hcp Ir‐Ni nanoplates is shown in **Figure** **1**a. Transmission electron microscopy (TEM) was used to confirm the hexagonal plate shape of hcp Ir‐Ni (Figure S2a, Supporting Information). The Ir content of hcp Ir‐Ni was 9.9 at%, as demonstrated using energy‐dispersive X‐ray spectroscopy (EDX) (Figure S2b, Supporting Information). EDX mapping revealed that hcp Ir‐Ni presented core–shell structure, wherein Ir was primarily distributed in the shell and Ni was distributed in the core (Figure 1b). Line‐scanning experimental results demonstrate the successful synthesis of core–shell hcp Ir‐Ni (Figure S3, Supporting Information). TEM images were used to determine that the thickness of the Ir shell ranged between 0.6 and 0.8 nm (Figure S4, Supporting Information). Powder X‐ray diffraction (PXRD) was used to evaluate the crystal structure of hcp Ir‐Ni. The absence of the characteristic peaks for the fcc Ni phase (JCPDS No. 04–0850, Fm3m) and the presence of the characteristic peaks for the hcp Ni (JCPDS No. 45–1027, P6_3_/mmc) and fcc Ir phases (JCPDS No. 46–1044, Fm3m) in the XRD pattern of hcp Ir‐Ni (Figure S5, Supporting Information) confirmed the purity of metastable hcp Ir‐Ni.

**Figure 2:**
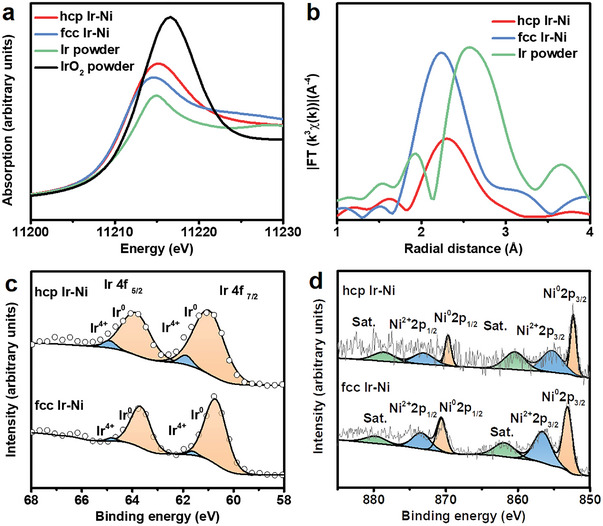
a) Normalized XANES spectra of hcp Ir‐Ni, fcc Ir‐Ni, Ir powder, and IrO_2_ powder recorded at the Ir *L*‐edge. b) Fourier transforms of Ir *L*‐edge EXAFS spectra of hcp Ir‐Ni, fcc Ir‐Ni, and Ir powder. c) XPS analyses of Ir 4f of hcp Ir‐Ni and fcc Ir‐Ni. d) XPS analyses of Ni 2p of hcp Ir‐Ni and fcc Ir‐Ni.

Spherical‐aberration‐corrected high‐angle annular dark field–scanning transmission electron microscopy (HAADF–STEM) was used to thoroughly evaluate the atomic distributions of Ir and Ni along different directions. A hexagonal array of Ir atoms along the [001] direction was observed in the first and the second layers (Figure 1c–e). The high‐resolution TEM images of a hcp Ir‐Ni nanoplate (Figure 1f,g) revealed that the atomic packing mode along the [100] direction was ABABAB, which was in agreement with the unique hcp crystal phase (Figure S1a2, Supporting Information and Figure 2h). Therefore, we concluded that hexagonal phase (P6_3_/mmc) Ir shells were grown onto hcp Ni cores.

Because fcc is the stable phase of Ir and Ni, fcc Ir‐Ni was then prepared by directly annealing hcp Ir‐Ni at 400 °C in a tube furnace under a 5% H_2_/95% Ar gas atmosphere. According to its XRD pattern, fcc Ir‐Ni comprised fcc Ni (JCPDS No. 04–0850) and fcc Ir phases (JCPDS No. 46–1044) and no hcp Ni phase (JCPDS No. 45–1027) (Figure S6a, Supporting Information). The morphology of fcc Ir‐Ni did not change considerably after calcination (Figures S6b,c). Spherical‐aberration‐corrected HAADF–STEM and fast Fourier transform analyses further demonstrated the successful preparation of fcc Ir‐Ni (Figure S6d–f, Supporting Information).

Direct synthesis of metastable phases is challenging because the formation energy of metastable phases is higher than that of stable phases. Therefore, it is critical to perform control experiments to evaluate the effect on the formation of metastable hcp structures. First, we used IrBr_3_, Ir(acac)_3_, and Ir(Ac)_3_ instead of IrCl_3_ as the metal precursor and maintained the other experimental conditions unchanged. The particles fabricated using IrBr_3_, Ir(acac)_3_, and Ir(Ac)_3_ were irregular (Figure S7a–c, Supporting Information). Moreover, the XRD results revealed that the crystal structure of Ni was fcc (JCPDS No. 04–0850) (Figure S7d, Supporting Information). Next, we explored the effect of reaction time on the properties of hcp Ir‐Ni (Figure S8, Supporting Information). After rapid heating for 0.5 h, hcp Ni particles with irregular shape were present in the product. Upon increasing reaction time, the particle size of the product became increasingly uniform. At a reaction time of 4 h, the product no longer comprised particles and exhibited hexagonal plate‐like morphology.

X‐ray absorption near‐edge structure spectroscopy (XANES) is highly sensitive to the electronic structure of 5d transition metals (TMs).^[^ ^22^, ^23^, ^24^ ^]^ The spectral features of hcp Ir‐Ni, fcc Ir‐Ni, IrO_2_, and Ir powder at the Ir *L3* edge are shown in **Figure** **2**a. The white lines in the XANES profiles of hcp Ir‐Ni, fcc Ir‐Ni, and Ir powder were located at approximately the same energy and were downshifted by 1.6 eV relative to the white line of IrO_2_. This demonstrates that the oxidation state of Ir^4+^ ions was comparable to those of fcc Ir‐Ni and Ir powder. Extended X‐ray absorption fine structure (EXAFS) spectroscopy is a sensitive analysis method for the local environment of TM ions.^[^ ^25^, ^26^ ^]^ The Fourier transform k3*χ*(k) functions of hcp Ir‐Ni and fcc Ir‐Ni are shown in Figure 2b. The peak at ≈2.6 Å in the EXAFS spectra of hcp Ir‐Ni and fcc Ir‐Ni was attributed to Ir–Ir bonds. Considering that the atomic radius of Ni is smaller than that of Ir, the primary peaks at ≈2.4 and ≈2.3 Å in the EXAFS spectra of hcp Ir‐Ni and fcc Ir‐Ni were attributed to the Ir‐Ni bonds of hcp Ir‐Ni and fcc Ir‐Ni, respectively. These values, which were considerably smaller than the length of the Ir–Ir bonds for Ir powder, were attributed to the different crystal structures of hcp Ir‐Ni and fcc Ir‐Ni resulting in different coordination environments. Because electrocatalysis is a surface‐sensitive reaction, a thorough understanding of the catalyst surface environment is critical. X‐ray photoelectron spectroscopy (XPS) was used to characterize the surface chemical states of hcp Ir‐Ni. The peaks at 60.87 and 61.62 eV in the Ir 4f XPS profile of hcp Ir‐Ni were attributed to Ir^0^ and Ir^4+^, respectively (Figure 2c). The binding energies of these peaks were higher than those of the corresponding peaks in the Ir 4f XPS profile of fcc Ir‐Ni. This was attributed to the strong electron synergistic effect of hcp Ir and Ni.^[^ ^27^ ^]^ A similar tendency was observed in Ni 2p XPS profiles. The binding energies of the peaks of Ni^0^ (853.20 eV) and Ni^2+^ (856.72 eV) and satellite peak (862.14 eV) in the Ni 2p XPS profile of fcc Ir‐Ni were higher than those of the corresponding peaks in the Ni 2p XPS profile of hcp Ir‐Ni (Figure 2d).^[^ ^28^ ^]^ The primary chemical state of Ir in fcc Ir‐Ni and hcp Ir‐Ni was metallic. The binding energy of Ir for hcp Ir‐Ni was 0.8 eV lower than that for fcc Ir‐Ni, indicating that more electrons were transferred from Ni to Ir in hcp Ir‐Ni than in fcc Ir‐Ni.

**Figure 3:**
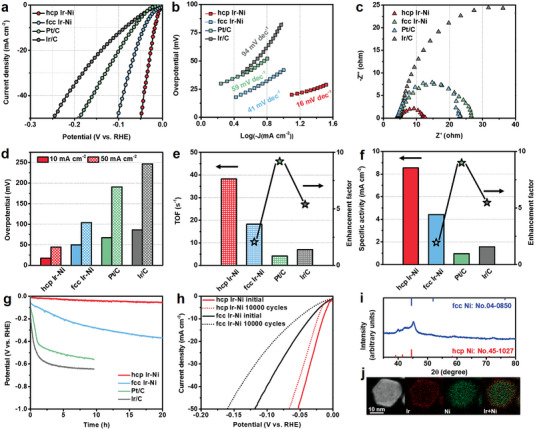
Activity evaluation in alkaline hydrogen evolution reaction. a) HER polarization plots and b) Tafel plots of hcp Ir‐Ni, fcc Ir‐Ni, Pt/C, and Ir/C. c) EIS Nyquist plots of hcp Ir‐Ni, fcc Ir‐Ni, Pt/C, and Ir/C. d) Overpotentials at current densities of 10 and 50 mA cm^−2^ of hcp Ir‐Ni, fcc Ir‐Ni, Pt/C, and Ir/C. e) TOF values of hcp Ir‐Ni, fcc Ir‐Ni, Pt/C, and Ir/C at the overpotential of 70 mV. f) The specific activity of hcp Ir‐Ni, fcc Ir‐Ni, Pt/C, and Ir/C at the overpotential of 70 mV. g) CP curves of hcp Ir‐Ni, fcc Ir‐Ni, Pt/C, and Ir/C at 10 mA cm^−2^. h) The polarization curves of hcp Ir‐Ni and fcc Ir‐Ni before and after continuous. i) XRD pattern of hcp Ir‐Ni after the stability test. j) HAADF‐STEM image and EDS mapping of hcp Ir‐Ni after the stability test.

To explore the catalytic applications of hcp Ir‐Ni, we analyzed its HER activity in an alkaline electrolyte. Hcp Ir‐Ni was loaded onto carbon powder and used as the electrocatalyst for the HER. Commercial Pt/C (20 wt.%), commercial Ir/C (20 wt.%), fcc Ir‐Ni, hcp Ni, and fcc Ni were selected as reference catalysts and were used for comparison (Figure S9, Supporting Information). All potentials were calibrated by 95% internal resistance (iR) compensation and adjusted to the RHE (Figure S10, Supporting Information). **Figure** **3**a shows the polarisation curves of hcp Ir‐Ni, fcc Ir‐Ni, Pt/C, and Ir/C in 1.0 M KOH obtained using linear sweep voltammetry. Hcp Ir‐Ni exhibited the best HER activity among the analyzed electrocatalysts. At the current densities of 10 and 50 mA cm^−2^ (denoted as *η*10 and *η*50, respectively), the overpotentials of hcp Ir‐Ni were only 17 and 48 mV, respectively (Figure 3d), whereas those of fcc Ir‐Ni were 42 and 104 mV, respectively. These results suggest the high HER activity of metastable Ir. The HER activity of hcp Ir‐Ni was also superior to those of Pt/C (67 mV at *η*10 and 191 mV at *η*50) and Ir/C (86 mV at *η*10 and 247 mV at *η*50). To determine the origin of the high activity of hcp Ir‐Ni, we also evaluated the HER activity of pure hcp Ni and fcc Ni. The overpotentials of hcp Ni at *η*10 and *η*50 were 153 and 257 mV, respectively, whereas those of fcc Ni were 212 and 302 mV, respectively (Figure S11, Supporting Information). These results indicate that hcp Ir presented abundant active sites for the HER. We also tested the effect of the Ir content on the catalytic performance of hcp Ir‐Ni (Figures S12–S14, Supporting Information). The results demonstrate that the HER activity of hcp Ir‐Ni with an Ir content of 9.9 at% was higher than those of hcp Ir‐Ni with Ir contents of 4.4, 16.2, and 12.0 at% (43, 27, and 36 mV, respectively at *η*10). The XRD results revealed the formation of fcc Ir‐Ni with increasing Ir content, which presented poorer HER performance than hcp Ir‐Ni.

The kinetics of the electrocatalytic HER were evaluated using the Tafel slopes of the electrocatalysts. The Tafel slope of hcp Ir‐Ni (16 mV dec^−1^) was considerably lower than those of fcc Ir‐Ni (41 mV dec^−1^), Pt/C (59 mV dec^−1^) and Ir/C (94 mV dec^−1^) (Figure 3b). The lowest Tafel slope of hcp Ir‐Ni indicates the critical role of hcp Ir in promoting the kinetics of the HER.^[^ ^29^, ^30^ ^]^ The charge‐transfer resistance (at −0.02 V vs RHE) of hcp Ir‐Ni was considerably smaller than those of fcc Ir‐Ni, Pt/C, and Ir/C (Figure 3c). This further indicates that the kinetics of the HER over hcp Ir‐Ni was faster than those over fcc Ir‐Ni, Pt/C, and Ir/C. To further evaluate the intrinsic activity of the metastable active sites of hcp Ir‐Ni, the TOFs of the electrocatalysts were calculated using their electrochemically active surface areas (ECSAs), and the results are shown in Figure S15, Supporting Information (Calculation details are included in the Experimental section).^[^ ^31^, ^32^ ^]^ The TOF of hcp Ir‐Ni at the overpotential of 70 mV (38.26 s^−1^) was higher than those of fcc Ir‐Ni (18.25 s^−1^), Pt/C (4.17 s^−1^), and Ir/C (7.07 s^−1^) (Figure 3e). This further demonstrates that the crystal phase of the catalyst promoted fast H_2_ evolution during electrocatalysis. We also evaluated the specific activity of the catalysts using their ECSA values. At the overpotential of 70 mV, the specific activity of hcp Ir‐Ni (8.55 mA cm^−2^) was 2.01, 9.0, and 5.48 times higher than those of fcc Ir‐Ni (4.41 mA cm^−2^), Pt/C (0.95 mA cm^−2^), and Ir/C (1.56 mA cm^−2^), respectively (Figure 3f). Therefore, the overall catalytic performance of hcp Ir‐Ni was superior to those of Pt/C and Ir/C. These results confirm that hcp Ir‐Ni is one of the best catalysts for the HER in alkaline media (Tables S2 and S3, Supporting Information).

Considering the thermodynamic instability of metastable phase materials, it is critical to evaluate the stability of metastable catalysts during reactions. The activity of hcp Ir‐Ni did not change considerably during a chronopotentiometry test (Figure 3g). The overpotential of hcp Ir‐Ni at *η*10 increased by only 40 mV after 10 h of testing, suggesting the excellent long‐term durability of hcp Ir‐Ni for the HER. In contrast, the overpotentials of Pt/C and Ir C at *η*10 decreased by approximately 500 and 530 mV, respectively, after 10 h of stability testing. The stability of the catalysts was also evaluated using cyclic voltammetry, and the polarisation curves before and after continuous potential scanning are shown in Figure 3h. After 10 000 cycles, the polarisation curves of hcp Ir‐Ni presented a small shift of ≈8 mV at *η*10. In contrast, fcc Ir‐Ni exhibited poor long‐term durability with a considerable increase in overpotential of 19 mV under the same experimental conditions. The XRD results demonstrate that the metastable hcp phase did not change during the stability test (Figure 3i). In addition, the HAADF–STEM and EDX mapping results revealed that the core–shell structure of hcp Ir‐Ni did not change after the stability test (Figure 3j). The high‐resolution TEM images of hcp Ir‐Ni after electrochemical testing (Figure 16a, Supporting Information) revealed that the local structure of hcp Ir was retained. Moreover, the Ir content of hcp Ir‐Ni after the stability test was determined to be approximately 9.1 at% using EDX (Figure 16b, Supporting Information). These results demonstrate the high stability of metastable Ir in hcp Ir‐Ni.

**Figure 4:**
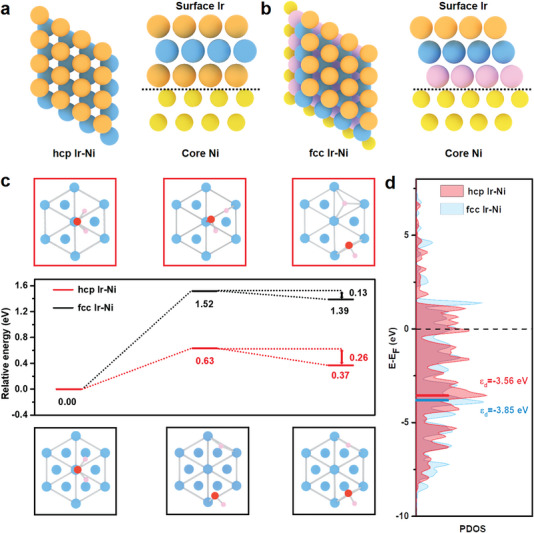
Theoretical simulations of HER processes. Top view and side view of a) hcp Ir‐Ni and b) fcc Ir‐Ni models, where the bottom two‐layer Ni are fixed while the surface Ir layers are fully optimized. c) Comparison of water dissociation kinetics on hcp Ir‐Ni and fcc Ir‐Ni. d) Projected density of states of surface Ir atoms.

To better understand the remarkable HER catalytic performance of hcp Ir‐Ni, we performed density functional theory calculations to thoroughly analyze the HER and its underlying electronic mechanism. Based on the TEM results, we cleaved five fcc Ni (111) and hcp Ni (0001) layers and substituted the Ni atoms in the top three layers with Ir atoms to build fcc Ir‐Ni and hcp Ir‐Ni models, respectively (**Figures** **4**a,b, respectively). In alkaline media, H_2_O molecules are the proton source for the HER, and the dissociation of O–H bonds determines the HER activity. Herein, we compared the energy barriers for the H_2_O dissociation reaction over hcp Ir‐Ni and fcc Ir‐Ni. Our results demonstrate that the kinetic energy barrier of hcp Ir‐Ni for H_2_O dissociation into *O and *OH (0.63 eV) was lower than that of fcc Ir‐Ni (1.52 eV) (Figure 4c). The d‐band center of the Ir atoms for hcp Ir‐Ni (−3.56 eV) was higher than that for fcc Ir‐Ni (−3.85 eV) (Figure 4d). Similar to the results of our previous study,^[^ ^33^ ^]^ the upshift in d‐band center was responsible for stabilizing the adsorption of reaction intermediates and accelerating the H_2_O dissociation reaction. Moreover, once the *H and *OH species were adsorbed, they were likely to recombine into H_2_O over fcc Ir‐Ni with a low energy barrier of 0.13 eV. In contrast, the adsorbed *H species were more stable over hcp Ir‐Ni with a higher recombination barrier of 0.26 eV (Figure 4c). Therefore, the strong H_2_O adsorption and fast H_2_O dissociation over hcp Ir‐Ni contributed to the remarkable HER activity of hcp Ir‐Ni in alkaline media. To confirm the simulation results, we also provide the geometry (initial, transition, and final states) of H_2_O dissociation over hcp Ir‐Ni and fcc Ir‐Ni (Figure S17, Supporting Information).

## 3 Conclusion

To summarize, metastable hcp Ir was synthesized for the first time. Owing to the unique crystal structure of hcp Ir, the intrinsic catalytic activity of metastable hcp Ir was high. Consequently, the HER performance of hcp Ir‐Ni was superior to those of fcc Ir‐Ni and commercial Pt/C electrocatalysts. Our findings demonstrate the critical role of phase‐engineering of noble‐metal‐based electrocatalysts for remarkable catalytic performance.

## Conflict of Interest

The authors declare no conflict of interest.

## Supporting information

Supporting Information

## Data Availability

The data that support the findings of this study are available from the corresponding author upon reasonable request.
