# Supplementary material for: Homogeneous Metastable Hexagonal Phase Iridium Enhances Hydrogen Evolution Catalysis
Source: Adv Sci (Weinh). 2023 Feb 12;10(11):2206063. doi: 10.1002/advs.202206063 (PMC10104624; doi:10.1002/advs.202206063)
Supplement: Supplementary file 1 — Supporting Information [file ADVS-10-2206063-s001.pdf]

Supporting information for

Homogeneous Metastable Hexagonal Phase Iridium Enhances Hydrogen Evolution Catalysis

*Shize Geng=, Yujin Ji=, Jiaqi Su=, Zhiwei Hu, Miaomiao Fang, Dan Wang, Shangheng Liu, Ling Li, Youyong Li, Jin-Ming Chen, Jyh-Fu Lee, Xiaoqing Huang and Qi Shao**

S. Geng, J. Su, M. Fang, S. Liu, L. Li, Prof. Q. Shao

College of Chemistry, Chemical Engineering and Materials Science, Soochow University Jiangsu, 215123, China.

E-mail: qshao@suda.edu.cn

S. Geng

College of Energy, Xiamen University, Xiamen, 361102, P. R. China

Dr. Y. Ji, Prof. Y. Li

Institute of Functional Nano & Soft Materials (FUNSOM), Soochow University, Jiangsu 215123, P. R. China.

Prof. Z. Hu

Max Planck Institute for Chemical Physics of Solids, Nothnitzer Strasse 40, Dresden 01187, Germany.

D. Wang

College of Energy, Soochow University, Jiangsu, 215123, P. R. China.

Dr. J. Chen, Dr. J. Lee

National Synchrotron Radiation Research Center, 101 Hsin-Ann Road, Hsinchu 30076, Taiwan.

Prof. X. Huang

State Key Laboratory of Physical Chemistry of Solid Surfaces, College of Chemistry and Chemical Engineering, Xiamen University, Xiamen, 361005, China.

S. Geng, Y. Ji, and J. Su are equally contributed.

Experimental section:

Chemicals. Nickel (II) formate dihydrate ($Ni(HCO_2)_2 \cdot 2H_2O$) and Iridium (III) chloride ($IrCl_3$) were purchased from Alfa Aesar. Glucose ($C_6H_{12}O_6$), ethanol and isopropanol were purchased from purchased from Sinopharm Chemical Reagent Co. Ltd. (Shanghai, China). Oleylamine ($CH_3(CH_2)_7CHCH(CH_2)_7CH_2NH_2$, 68%-70%) was purchased from J&K. Nafion was purchased from Aldrich. Commercial Pt/C (20 wt%) and commercial Ir/C (20 wt%) were purchased from Johnson Matthey (JM) Corporation. All the chemicals were used as received without further purification. The water was processed through an ultra-pure purification system (Aqua Solutions).

Synthesis of hcp Ir-Ni. $Ni(HCO_2)_2 \cdot 2H_2O$ (0.05 mmol, 9.2 mg), $IrCl_3$ (0.01 mmol, 3 mg), 330 mg glucose, and 5.0 mL oleylamine were mixed into a 35 mL glass vial. The mixture was ultrasonicated for 2 h, then heated at 230 °C for 5 h. After natural cooling, the product was washed with a mixture solution (Vcyclohexane : Vethanol = 1 : 8) for three times, then dried at 80 °C for 3 h.

Synthesis of hcp Ni and fcc Ni. $Ni(HCO_2)_2 \cdot 2H_2O$ (0.05 mmol, 9.2 mg), 330 mg glucose, and 5.0 mL oleylamine were mixed into a 35 mL glass vial. The mixture was ultrasonicated for 2 h, then heated at 230 °C for 5 h. After natural cooling, the product was washed with a mixture solution (Vcyclohexane : Vethanol = 1 : 8) for three times, then dried at 80 °C for 3 h.

The similar procedure was adopted for the preparation of fcc Ni except adding glucose at the beginning.

Characterization. Crystal structure of the catalysts were characterized by X-ray powder diffraction (XRD, Philips X'pert PRO MPD diffractometer) with a Cu Kα radiation source (λ = 0.15406 nm). Transmission electron microscopy (TEM), high-angle annular dark-field scanning transmission electron microscopy (HAADF-STEM) and STEM energy-dispersive X-ray spectroscopy (HAADF-STEM-EDX) were conducted on a FEI Tecnai F20 TEM at an

accelerating voltage of 200 kV. The morphology and size of the nanocrystals were determined by TEM (Hitachi, HT7700) at 120 kV. XAS data were collected at the TLS-07A beamline of the National 14 Synchrotron Radiation Research Center (NSRRC, Hsinchu, Taiwan) (Ir powder as reference standard sample). The EXAFS data were processed according to the standard procedures using the ATHENA module implemented in the “IFEFFIT software packages” in the method section of the revised manuscript. XPS experiment was carried out on the SSI S-Probe XPS Spectrometer. The carbon peak at 284.8 eV was used as a reference to correct for charging effect.

Electrochemical measurements. To prepare the catalysts for HER, the hcp Ir-Ni were subjected to thermal annealing at 250 ºC in H_2 gas for 1 h before test. The electrochemical measurements were carried out on CHI 660E workstation. All the experiments were carried out at room temperature. Electrochemical measurements were evaluated in a three-electrode setup with a saturated calomel electrode as the reference electrode and a carbon electrode as the counter electrode, a glassy carbon (GC) disk electrode (5 mm in diameter) was used as the working electrode. In this paper, 2 mg catalyst and 0.4 mL isopropanol were treated by ultrasound for 30 min, then 5 μL naphthol was added to prepare ink with different electrocatalysts, and then 20 μL ink was dripped into the glassy carbon electrode with a geometric area of 0.196 cm^2 to prepare the working electrode. We carried out the HER activity test on the glassy carbon electrode, and controlled the loading amount of noble metal constant (10 $\mu g_{noble\ metal}/cm^2$) for all the electrocatalysts. In the chronopotentiometry test, we also load 10 $\mu g_{Ir}/cm^2$ on glassy carbon electrode without rotated experiments. Linear sweep voltammetry (LSV) was recorded in 1.0 M KOH at a scan rate of 10 $mV\cdot s^{-1}$ to obtain the polarization curves. The CP tests were measured in 1.0 M KOH at a constant current density of 10 mA cm^{-2} with loading 10 $\mu g_{Ir}/cm^2$ catalyst on glassy carbon electrode.

Computational details. Theoretical simulations were conducted in VASP under the framework of DFT with the projector-augment plane wave basis set.[1-3] The exchange-correlation interaction adopted the description formula of Perdew-Burke-Ernzerhof within generalized gradient approximation.[4] Meanwhile, the Grimme method (DFT-D3) was adopted to correct the weak van der Waals interaction between water and metal surface.[5] For all calculations, the electronic cut-off energy was set to 400 eV and the convergence thresholds of energy and force during geometry optimizations were corresponding to $1E^{-4}$ eV and 0.02 eV $Å^{-1}$, respectively. As for the transition-state calculations, the combination of the climbing nudged elastic band method[6] and the improved dimer method[7] was used to search the transition state of water dissociation and determine the reaction and activation energy. The Brillouin zone was sampled in the Gamma-centered Monkhorst-Pack K-point 2×2×1 mesh for the surface model[8]. Besides, we applied a vacuum space larger than 15 Å to avoid the interactions between the two neighboring images.

Supplementary Figures and Tables: Figures S1-S17, Tables S1-S3 and Note S1

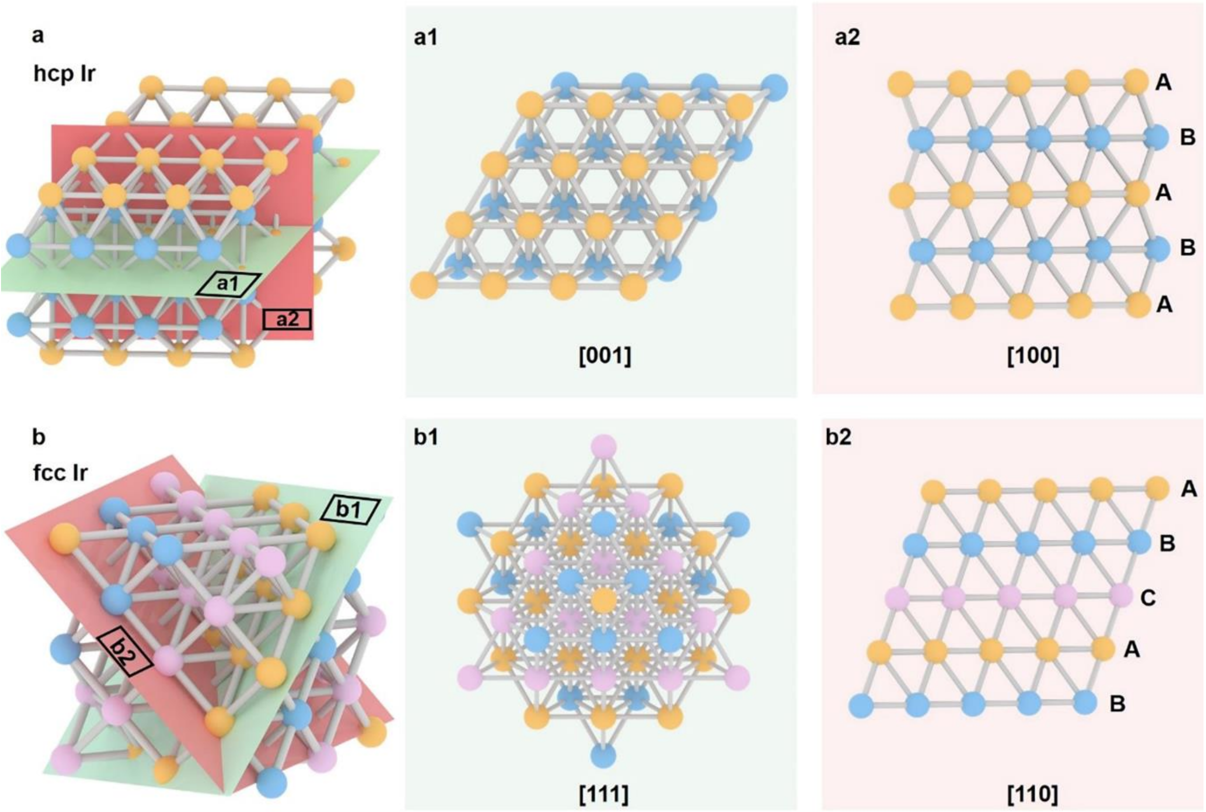

Figure S1. Schematic structures of hcp Ir and fcc Ir. (a) A crystal model of hcp Ir. The cross-section images of hcp Ir from (a1) [001] direction and (a2) [100] direction. (b) A crystal model of fcc Ir. The cross-section images of fcc Ir from (b1) [111] direction and (b2) [110] direction.

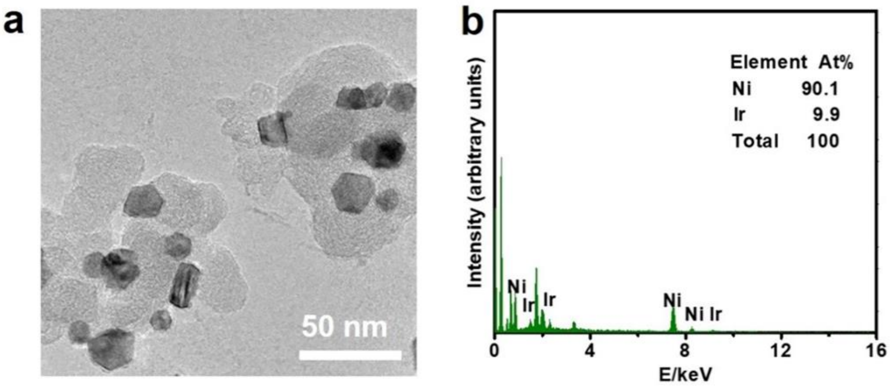

Figure S2. (a) TEM image and (b) EDX spectrum of hcp Ir-Ni.

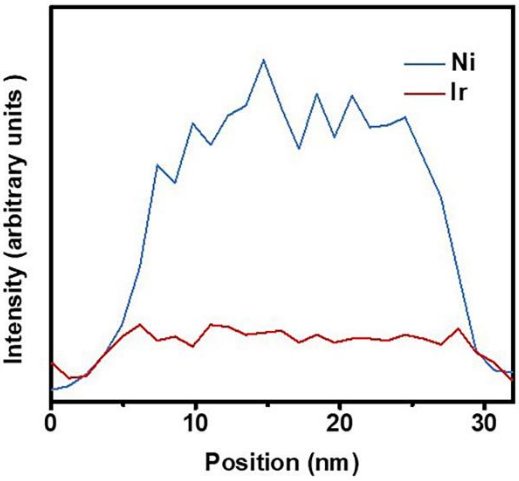

Figure S3. EDS line-scans of hcp Ir-Ni nanoplate.

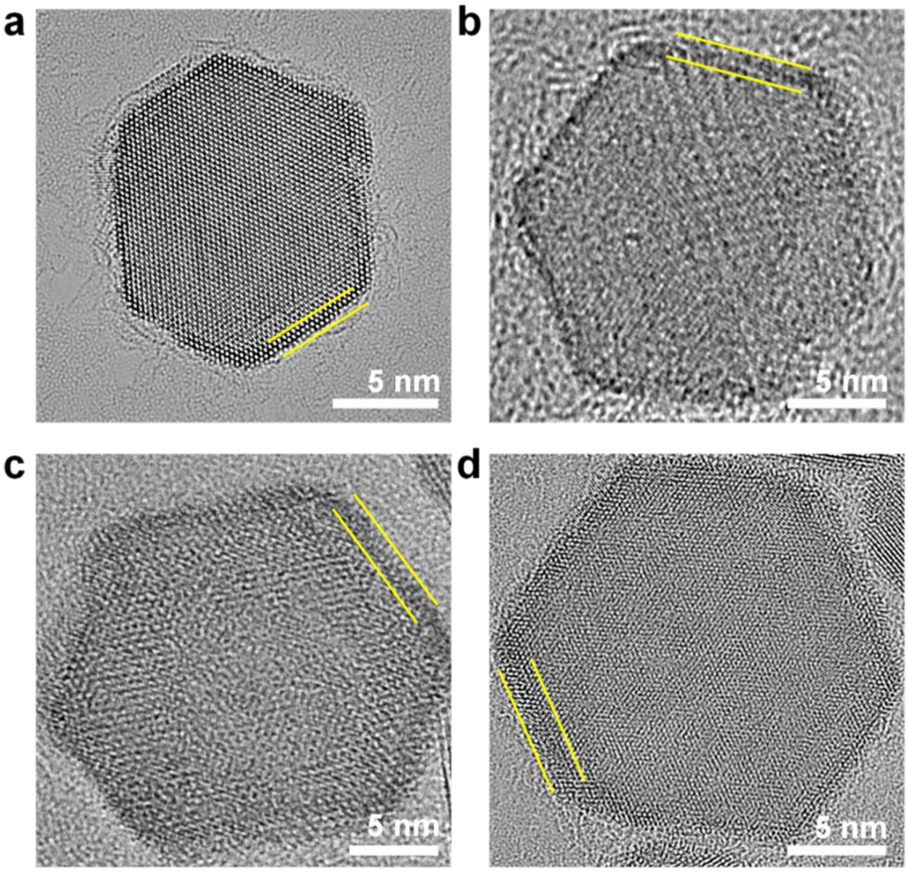

Figure S4. TEM images of hcp Ir-Ni (The yellow line marks the outer shell).

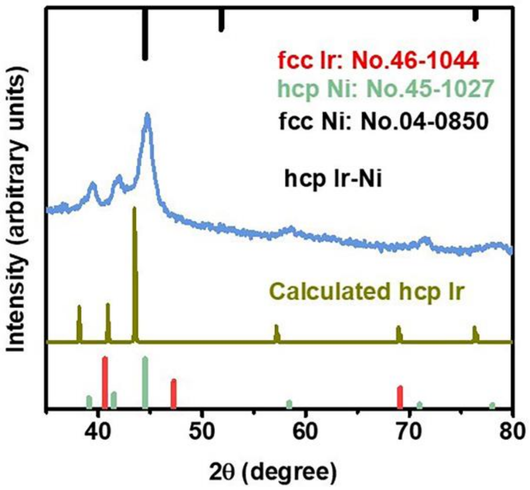

Figure S5. XRD pattern of hcp Ir-Ni. Brown line is the simulated XRD pattern of hcp Ir.

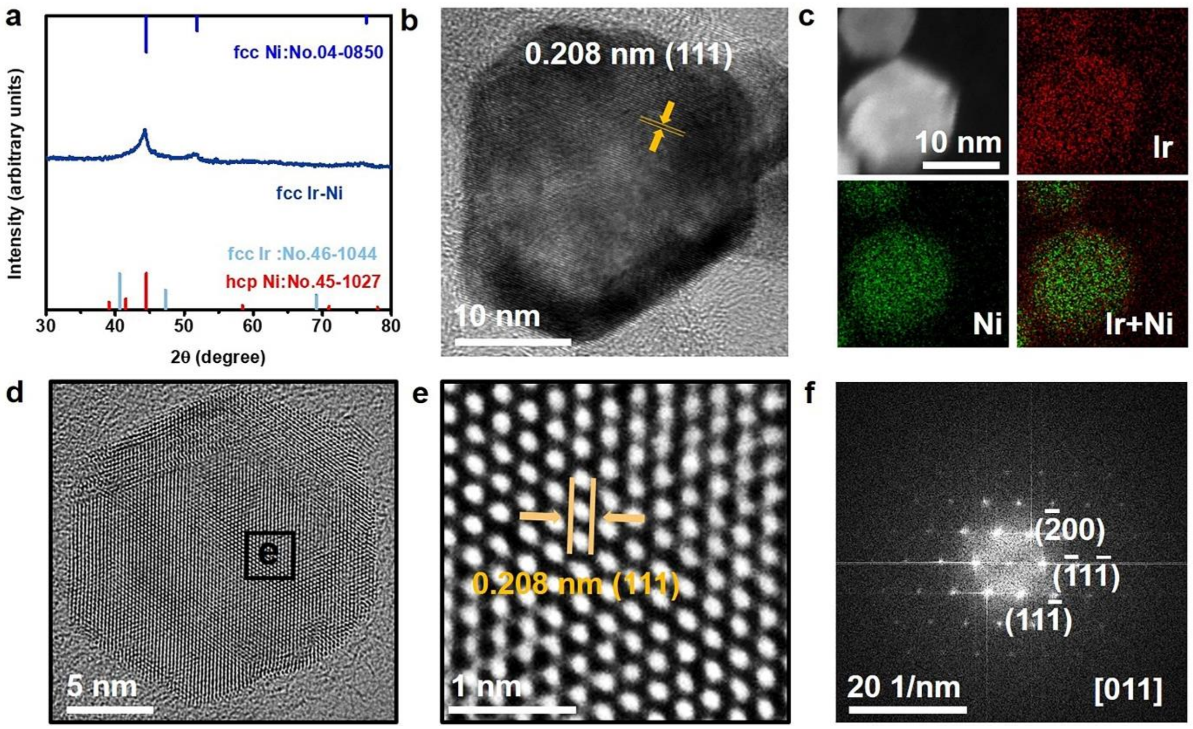

Figure S6. (a) XRD pattern, (b) HRTEM image and (c) HAADF-STEM image and EDX mapping of fcc Ir-Ni. (d) The spherical aberration corrected HAADF-STEM image of a fcc Ir-Ni nanoplate. (e) High-resolution HAADF image and (f) FFT image taken from the selected centre area (area e).

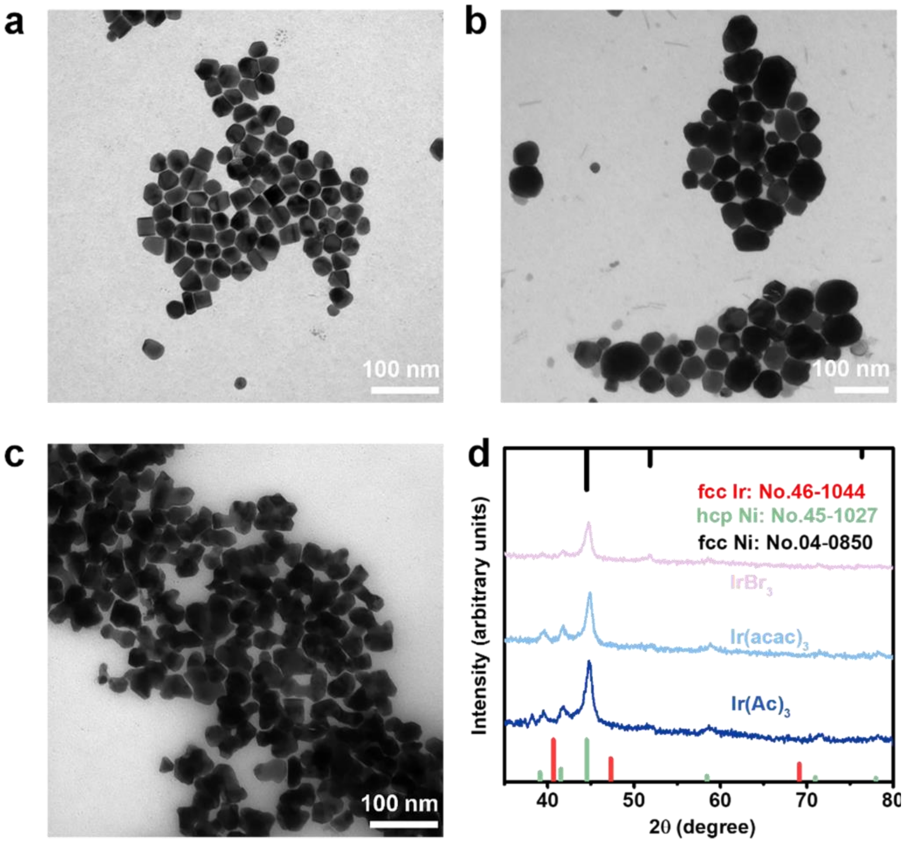

Figure S7. TEM images of the Ir-Ni compound when metal precursor changed from $IrCl_3$ to (a) $IrBr_3$, (b) $Ir(acac)_3$ and (c) $Ir(Ac)_3$. (d) XRD patterns of products synthesized from different metal precursors.

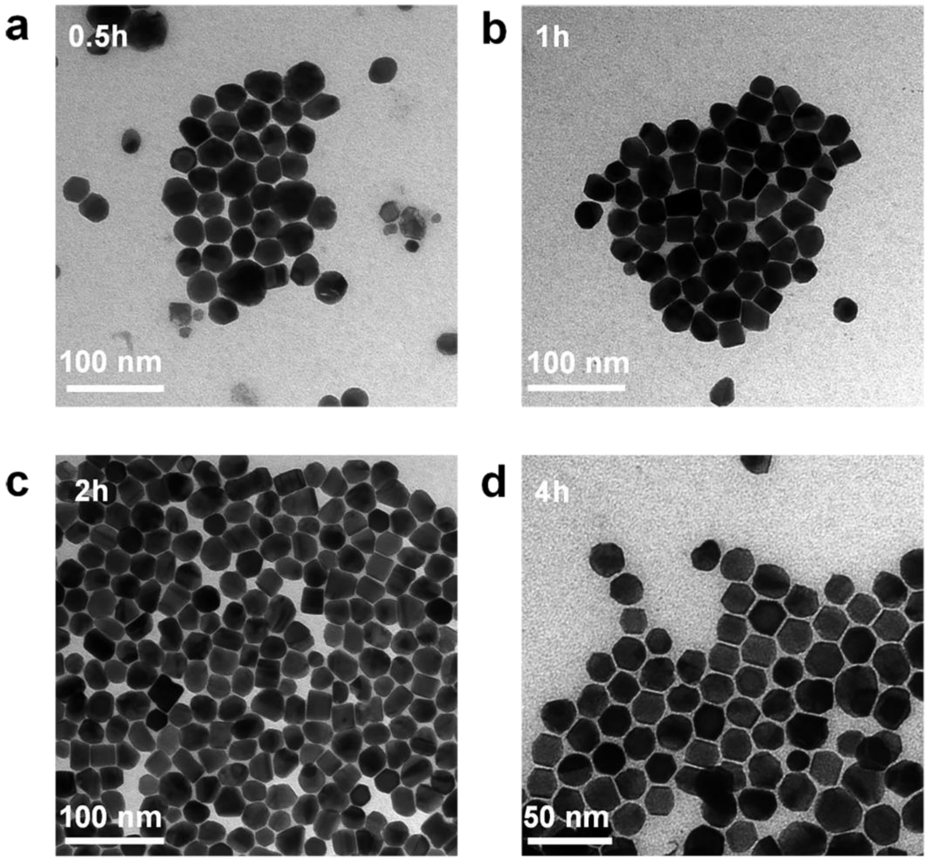

Figure S8. TEM images of hcp Ir-Ni synthesized from different time: (a) 0.5 h, (b) 1 h, (c) 2 h and (d) 4 h.

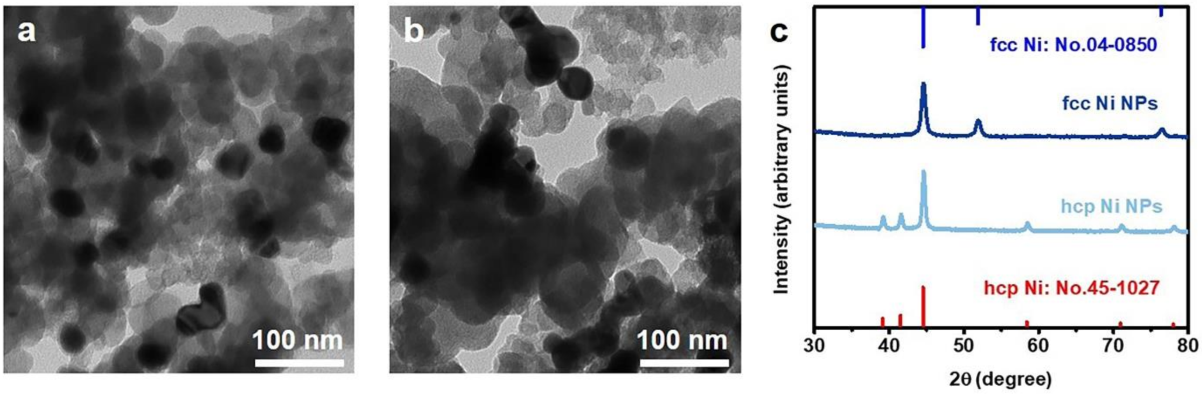

Figure S9. TEM images of (a) hcp Ni and (b) fcc Ni. (c) The related XRD patterns.

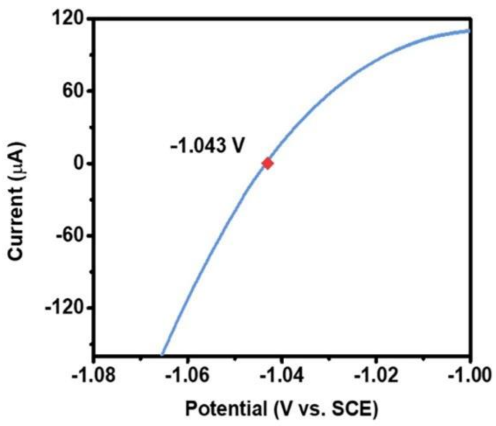

Figure S10. Current-potential curve for the calibration of saturated calomel electrode with respect to RHE in the highly pure H_2-saturated 1.0 M KOH by using a Pt wire as the working electrode.

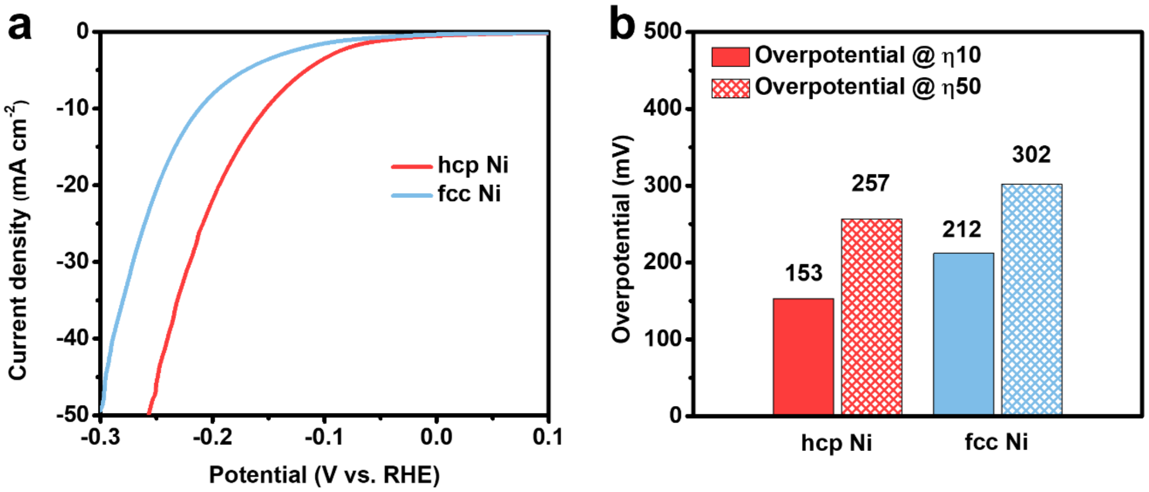

Figure S11. (a) HER performances of hcp Ni and fcc Ni. (b) The overpotentials of hcp Ni and fcc Ni at the current densities of 10 mA cm^{-2} and 50 mA cm^{-2}.

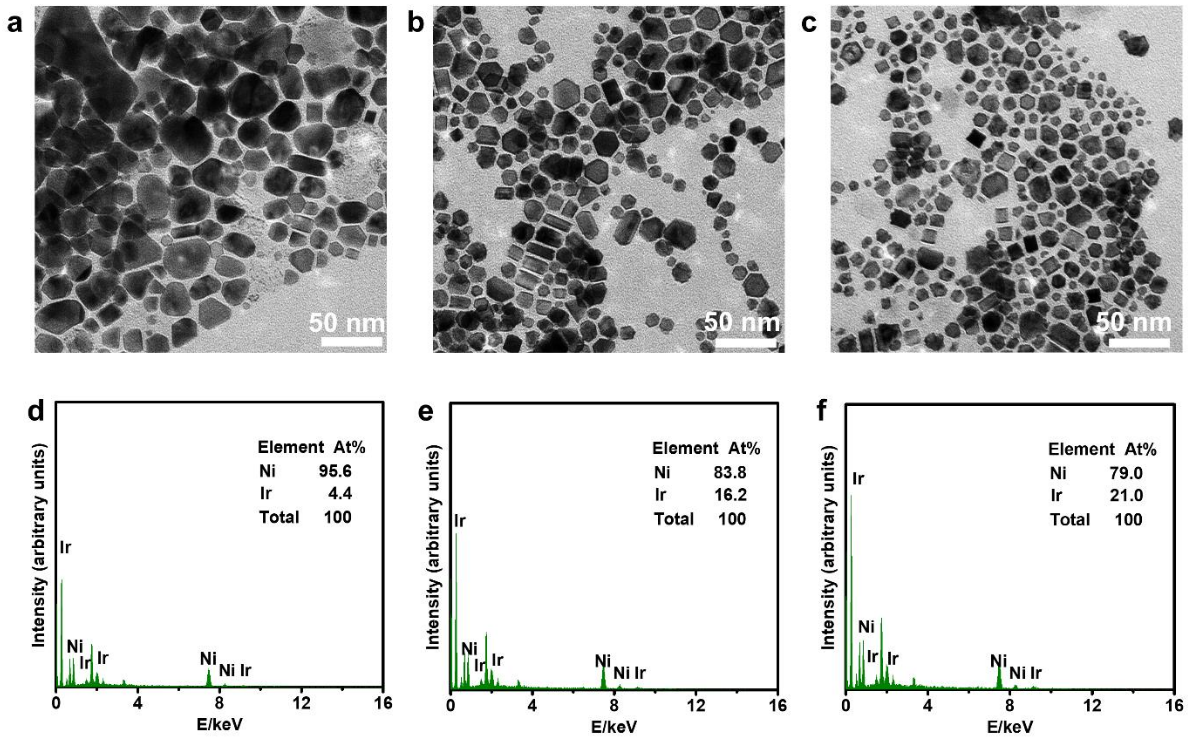

Figure S12. (a, b, c) TEM images and (d, e, f) EDX images of hcp Ir-Ni with different atomic ratios of iridium: (a, d) 4.4%, (b, e) 16.2% and (c, f) 21.0%.

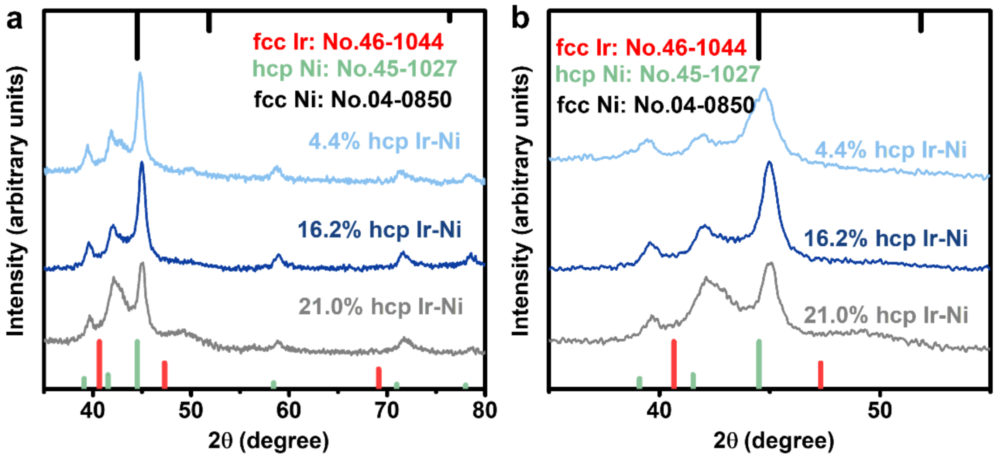

Figure S13. (a) XRD patterns and (b) the enlarged patterns of 4.4% hcp Ir-Ni, 16.2% hcp Ir-Ni and 21.0% hcp Ir-Ni.

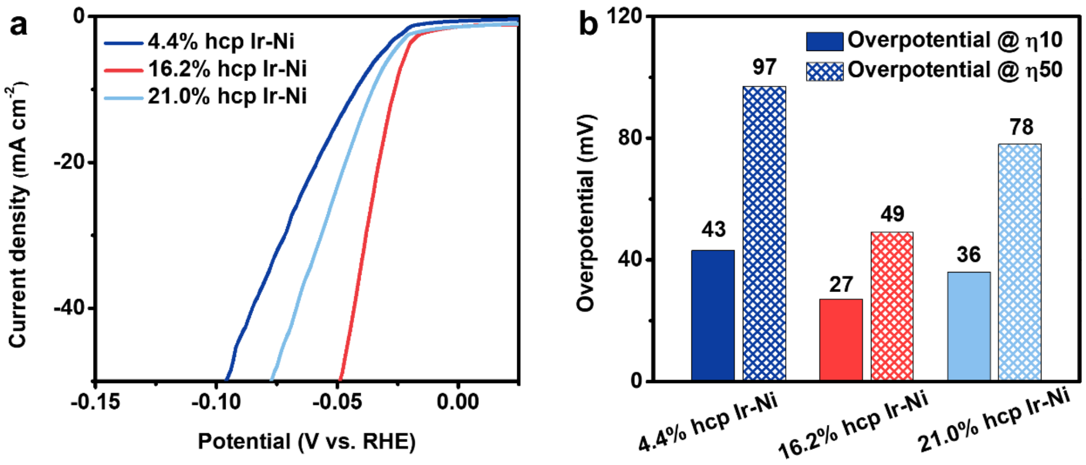

Figure S14 (a) LSV curves and (b) HER performances of 4.4% hcp Ir-Ni, 16.2% hcp Ir-Ni and 21.0% hcp Ir-Ni.

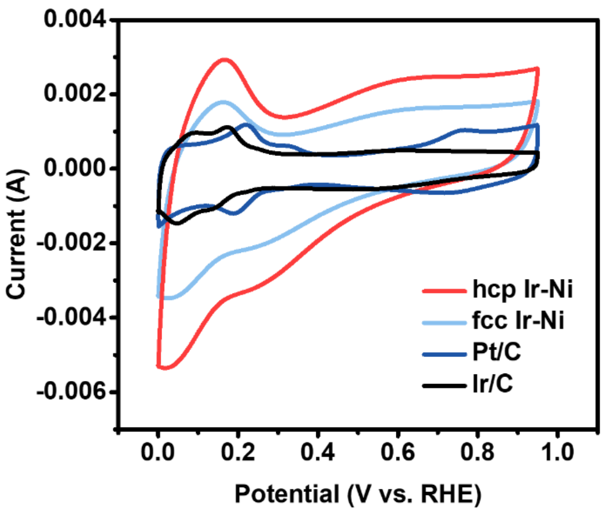

Figure S15. Cyclic voltammogram of hcp Ir-Ni, fcc Ir-Ni, Pt/C and Ir/C in 1.0 M KOH electrolyte obtained at the scan rate of 500 mV s^{-1}.

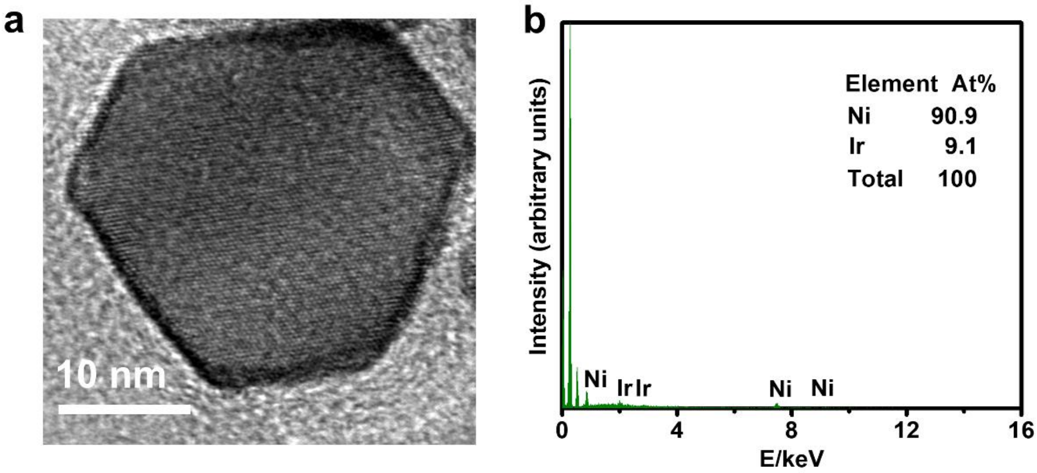

Figure S16. (a) HAADF-STEM image, (b) EDX image of hcp Ir-Ni after stability test.

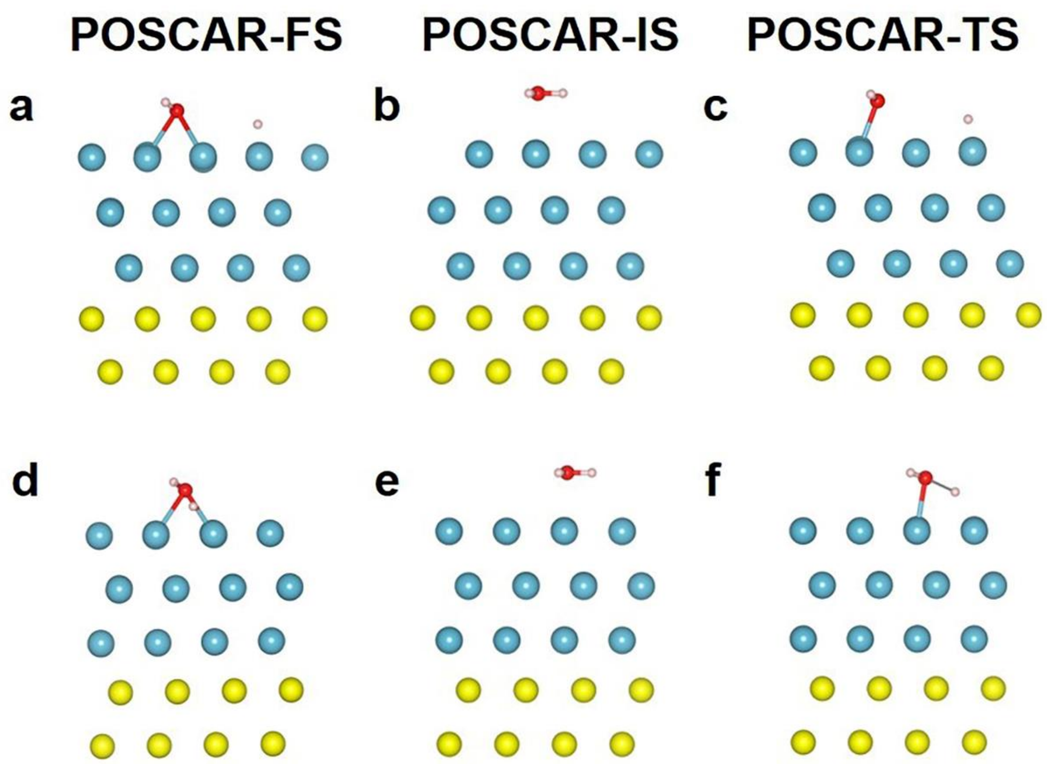

Figure S17. Initial state, transition state and final state of water dissociation on (a, b, c) fcc Ir-Ni and (d, e, f) hcp Ir-Ni.

Table S1. Crystallographic information for hcp Ni and fcc Ni.

Material	Crystal system	Bravais lattice	Unit-cell dimensions	Space group
**hcp Ni**	Hexagonal	Primitive	$a = b = 2.651$ Å, $c = 4.343$ Å; $\alpha = \beta = 90$ °, $\gamma = 120$ °	$P6_3/mmc$ (194)
**fcc Ni**	Cubic	Face-centered	$a = b = c = 3.523$ Å; $\alpha = \beta = \gamma = 90$ °	Fm3m (225)

Table S2. Comparisons of HER performance for various noble metal-based electrocatalysts.

Catalysts	Electrolyte	Substrates	**Overpotential (mV @ 10 mA cm^{-2})**	**Tafel slope (mV dec^{-1})**	Refs
hcp Ir-Ni	1 M KOH	GCE	**17**	**16**	This work
Ru@C_2N	1 M KOH	GCE	17	38	*Nat. Nanotechnol.* **2017**, *12*, 441-446.
Au-Ru NWs	1 M KOH	GCE	25	30.8	*Nat. Chem.* **2018**, *10*, 456-461.
Pt/NC	1 M KOH	GCE	46	36.8	*Nat. Commun.* **2020**, *11*, 1029.
Pt-Ni nano-multipods	0.1 M KOH	GCE	65	78	*Nat. Commun.* **2017**, *8*, 15131.
Ni-branched Pt-islands	0.1 M KOH	GCE	49	69	*J. Am. Chem. Soc.* **2019**, *141*, 16202-16207.
PtNi-O	1 M KOH	GCE	40	35.7	*J. Am. Chem. Soc.* **2018**, *140*, 9046-9050.
Ir@CoNC	1 M KOH	GCE	55	119	*Angew. Chem. Int. Ed.* **2019**, *58*, 11868-11873.
NiO_x/Pt_3Ni	1 M KOH	GCE	40	NA	*Angew. Chem. Int. Ed.* **2016**, *55*, 12859.
Pt_5/HMCS	1 M KOH	GCE	46.2	48.1	*Adv. Mater.* **2020**, *32*, 1901349.
Ru_2Ni_2	1 M KOH	GCE	40	23	*Nano Energy* **2018**, *47*, 1-7.
Dr-Pt	1 M KOH	GCE	26	52	*Adv. Mater.* **2022**, *34*, 2106973.
Pt/MOF-O	1 M KOH	GCE	66	101.6	*J. Am. Chem. Soc.*

			**2021**, *143*, 16512-16518.

Table S3. Comparisons of TOF values for various electrocatalysts in alkaline HER.

Catalyst	Electrolytes	TOF (s^{-1}) @ mV vs. RHE	Refs
**hcp Ir-Ni**<br>**fcc Ir-Ni**	**1 M KOH**<br>**1 M KOH**	**38.23 @ 70 mV**<br>**23.52 @ 70 mV**	**This work**
Au-Ru-2 NWs	1 M KOH	0.31 @ 50 mV	*Nat. Chem.* **2018**, *10*, 456-461.
Pt_1/N-C	1 M KOH<br>0.5 M H_2SO_4	1.89 @ 50 mV<br>22.07 @ 50 mV	*Nat. Commun.* **2020**, *11*, 1029.
Ni-branched Pt-islands	0.1 M KOH	35.11 @ 70 mV	*J. Am. Chem. Soc.* **2019**, *141*, 16202-16207.
Ni/CQDs	1 M KOH	5.03 @ 100 mV	*Angew. Chem. Int. Ed.* **2020**, *59*, 1718-1726.
SLNP	1 M KOH	3 @ 70 mV	*Adv. Mater.* **2020**, *32*, 1908521.

Note 1. Turnover frequency (TOF) calculation of Ir-hcp Ni, Ir-fcc Ni, Pt/C and Ir/C.
Turnover frequency calculated from the following equation:

$$\mathrm{TOF} = \frac{\upsilon}{\mathrm{n_{Pt}}}$$

Where υ : Reaction rate = $\frac{j}{zF}$ [mol s^{-1}]
j: current [A] obtained from LSV measurements, F: faraday constant [C moI^{-1}] and z= 2 as two electrons need to be transferred to produce H_2.
And n_{Pt} [mole]: mole of Pt active sites, calculated from the charge obtained by integrating the area of the hydrogen desorption peaks in the CV measurement.

References

[1] G. Kresse, J. Furthmüller, *Phys. Rev. B* **1996**, *54*, 11169-11185.

[2] G. Kresse, J. Furthmüller, *Comput. Mater. Sci.* **1996**, *6*, 15-50.

[3] G. Kresse, D. Joubert, *Phys. Rev. B* **1999**, *59*, 1758-1775.

[4] J. P. Perdew, K. Burke, M. Ernzerhof, *Phys. Rev. Lett.* **1996**, *77*, 3865-3868.

[5] J. Antony, B. Schmidt, C. Schütte, *J. Chem. Phys.* **2010**, *132*, 154104.

[6] G. Henkelman, H. Jónsson, *J. Chem. Phys.* **2000**, *113*, 9978-9985.

[7] G. Henkelman, H. Jónsson, *J. Chem. Phys.* **1999**, *111*, 7010-7022.

[8] H. J. Monkhorst, J. D. Pack, *Phys. Rev. B*, **1976**, *13*, 5188.
